# Live-in care for older people with a focus on those living with dementia: a scoping review

**DOI:** 10.1186/s12877-026-07983-w

**Published:** 2026-07-29

**Authors:** Morwenna Rogers, Ruth Bartlett, Anita Chonzi, Catherine Henderson, Stefan Brown, Laura Cole, Elisa Aguzzoli, George Rook, Phil Joddrell, Aimee Day, Jo Thompson Coon

**Affiliations:** 1 University of Exeter, Exeter, United Kingdom; 2 University of Southampton, Southampton, United Kingdom; 3 Care Policy and Evaluation Centre, London School of Economics and Political Science, London, UK; 4 Royal Holloway, University of London, Egham, UK; 5 University of West London, London, UK; 6 Living with Dementia and Activist Campaigner, Shrewsbury, UK; 7 Dementia UK, London, UK; 8 AD Dementia Advocacy, Brighton, UK

## Abstract

**Background:**

Research has shown that adults prefer to remain in their own homes as they age. Care homes are expensive and as the population ages, availability is likely to become more limited and unaffordable to many. Live-in care, when a paid worker moves into the care recipient’s home to provide support, offers a potential alternative to residential care. This is the first scoping review of research on live-in care with a particular focus on people living with dementia. It seeks to reframe understanding of long-term care options for people with dementia.

**Methods:**

This scoping review followed conduct guidelines as described by the JBI. Systematic searches were conducted using seventeen bibliographic databases and sources rich in grey literature for quantitative or qualitative studies investigating live-in care. Results were double screened by two independent reviewers. Data were extracted and checked. Common findings found during data extraction were translated into themes. A lay advisory group was also consulted as to which themes were most relevant to them.

**Results:**

Most studies were qualitative in nature with a focus on the experience of the live-in care worker. In all studies live-in care workers were from countries outside of where they worked. Common themes arising from the findings of the studies included: motivations for choosing live-in care; relationships between live-in carer and care recipient and their families; exploitation of care workers and perceived quality of the care provided. Only seven studies focused on live-in care for people with dementia. These results address a critical gap in knowledge about a global form of care.

**Conclusion:**

Live-in care offers a promising alternative to residential care. Existing studies highlight a range of issues that should be addressed with live-in care arrangements, including exploitation of the workforce, cultural differences between workers and the families, a lack of training for live-in care workers and the importance of good relationships with this model of care. This scoping review identifies a previously unrecognised pattern, namely a focus on the live-in care worker, rather than the experiences of those receiving care. More studies investigating live-in care specifically for people living with dementia would help inform whether this model of care is effective for them.

**Supplementary Information:**

The online version contains supplementary material available at https://doi.org/10.1186/s12877-026-07983-w.

## Background and rationale

The number of people aged 85 + years with high dependency and in need of 24-hour care will double by 2035 and of these, people with dementia form the largest diagnostic group [1]. Often, care homes are considered the only option for people with dementia when their care needs increase, and people with dementia are five times more likely to move into a care home than those without a dementia diagnosis [2]. This might be because care home placement is considered the most affordable option for local authorities and offer an all-encompassing care environment for those with more complex needs, or because families have reached ‘tipping point’ and no longer feel they can support a relative at home [3].

Research has shown that many older adults prefer to remain in their own homes and age in place [4]. ‘Ageing in place’ means ‘staying put’ for as long as possible as you grow older although the exact definition is disputed [5]. Not having to move home unless you become severely incapacitated is a common way of thinking about ageing in place [5].

Ageing in place could be facilitated by live-in care, which is when a paid care worker moves into a care recipient’s home to provide support with day-to-day tasks. It offers a potential alternative to a move to a care home, which could enable people with dementia to remain at home while providing breaks for family carers. Despite its potential to reduce costs and allow older people to age in their home settings, live-in care is a relatively unknown market and a rarely examined model of service delivery.

A scoping review published in 2017 [6] examined the perspectives of families and care recipients of live-in care workers although most studies were conducted in a single country (Israel). The review highlighted the diverse roles of live-in workers, family dynamics, conflicts and power negotiations however it did not focus on people with dementia or examine packages of care (a bundle of items or services provided to support people unable to fully care for themselves). Since then, there has been a growth of studies of live-in care from Europe, particularly from Germany, Switzerland and the UK.

The aim of this review is to identify and map current evidence on live-in care for older adults and people living with dementia, paying particular attention to data on how and whether this care model supports ageing in place for people living with dementia. For this review we use one of the themes of ‘ageing in place’ as described by Forsyth and Molinsky [5]: to ‘stay put’ for as long as possible.

The review addressed the following research questions:

1. What is the volume, range and nature of evidence on live-in care for older adults?
2. What is the volume, range and nature of evidence on whether live-in care supports ageing in place for older adults with dementia?

## Methods

This scoping review followed the updated methodological guidance for the conduct of scoping reviews from the JBI Scoping Review Methodology Group which draws on the Population-Concept-Context framework [7] and the protocol is available at https://zenodo.org/records/15690260. PRISMA-ScR was used to guide the reporting of this review.

### Eligibility criteria

Studies were included if they met the following criteria:

#### Population

Studies involving older adults or older adults living with dementia, who received a live-in care package.

#### Concept

Live-in care either privately funded or funded and arranged by local authorities, health agencies (e.g. NHS trusts in the UK) or care agencies, where live-in care involves a dedicated, care worker moving into the home to provide personal care and help with domestic tasks.

#### Context

Older person’s own home in any geographical location.

#### Study design

Any studies providing empirical evidence were included.

Studies were excluded if the care was provided communally, e.g. in retirement villages, if the live-in care was provided by family members or if domiciliary care was provided where the care worker did not live with the older person. Where older people populations were not distinguishable from other care recipients (e.g. people with disabilities or children with live-in nannies), studies were excluded. Studies were also excluded if there were no outcomes or experiences related to live-in care within the home, e.g. outcomes only relating to integration of migrant care workers into a new country.

### Search methods

The following databases were searched between 25th April and 2nd May 2025: Medline, APA PsycINFO, EMBASE, Social Policy and Practice and Health Management Information Consortium (all via Ovid), CINAHL and Ageline (via EBSCOhost), British Nursing Index, ASSIA, Sociology Database and Sociological Abstracts (via ProQuest), the Cochrane Database of Systematic Reviews, and the CENTRAL database (via the Cochrane Library), and the Social Science Citation Index (via Web of Science). The search used a combination of free text terms and subject headings to cover the concepts of live-in carers, older people and the home setting. No limitations on publication year or language were imposed.

The final database search strategies are available in Appendix 1.

Backwards and forwards citation searching of included papers was carried out and related reviews were examined for additional studies. No journal titles were identified as being pertinent to this topic and therefore no handsearching was carried out. Authors were contacted for more details where only abstracts were found.

### Grey literature

In addition to searching databases rich in grey literature (SPP and HMIC), conference abstract databases (via Web of Science) were searched as well as ProQuest Dissertations and Theses. Repositories of grey literature (the WHO Institutional Repository, WorldCat and CORE) were searched. Searches were also run on Google Scholar and the Bielefeld Academic Search Engine. For the search engines, the phrase “live in care” was searched and the first 100 results were reviewed.

The following websites were searched for relevant studies: Alzheimer’s Association (US), Alzheimer’s Society (UK), Alzheimer’s Disease International, Homecare Association (UK). We also searched the websites of county councils of England as existed in May 2025 by following internal links to care provision or publications.

### Study selection

All studies were imported into EndNote V21 for deduplication and then uploaded to Covidence for screening. After an initial test screen, reviewers (MR, JTC, AC) met to finalise inclusion/exclusion criteria to ensure consistent application. All titles and abstracts were assessed by two reviewers (MR or JTC or AC) independently, with disagreements resolved by discussion. The full texts of eligible studies were retrieved and reviewed in Covidence by two independent reviewers (MR or JTC or AC). Disagreements were resolved by discussion.

### Data extraction

The following details were extracted for each study: author details, country of publication, study design, aim of the study, characteristics of the live-in carers, characteristics of the care recipients, intervention details, outcome measures, inequalities using PROGRESS plus criteria [8], data collection method, type of analysis and themes or findings that informed the research question. Data were extracted by one reviewer (MR or JTC) and checked by a second (AC).

### Public involvement

The scoping review is part of a broader project about live-in care (https://fundingawards.nihr.ac.uk/award/NIHR163287). A lay advisory group was set up as part of the broader project. A workshop was held with the group on 4th September 2025 to review and suggest search terms used in the database searches and identify which topics relating to live-in care were most important to them. Additional terms (e.g. home aide and end-of-life care) were checked against the search following the workshop and a follow-up search was conducted in September 2025 for search terms that found additional studies matching the criteria (see Appendix 1). Topics identified by the Lay Advisory Group helped to inform the structure of the review findings.

### Collating, summarising and reporting the results

Key characteristics of all included studies were recorded including the country of study population studied (care recipients, live-in carers, family members or care providers), experiences relating to live-in care, characteristics of carers, comorbidities of care recipients and descriptions of care packages. Headings of themes or study findings were extracted into an Excel spreadsheet by one reviewer (MR or JTC) and checked by another (AC). Themes that were common across studies were tabulated. The lay advisory group also advised on themes that were important to them. Extracted themes that were relevant to the research question formed the themes addressed in this review. Examples of themes discussed in the papers but not covered by this review included the adjustment of migrant live-in carers to new lives abroad and the exploitation of workers, unless it related specifically to the effectiveness or experience of live-in care. Themes identified by the Lay Advisory Group as important, but which were not covered by the studies in relation to the research questions were the health and safety of care workers with regards to physical health e.g. heavy lifting and the effects of Brexit on the live-in care workforce.

Following the guidelines for scoping review methods, no critical appraisal was conducted.

## Results

**Fig. 1:**
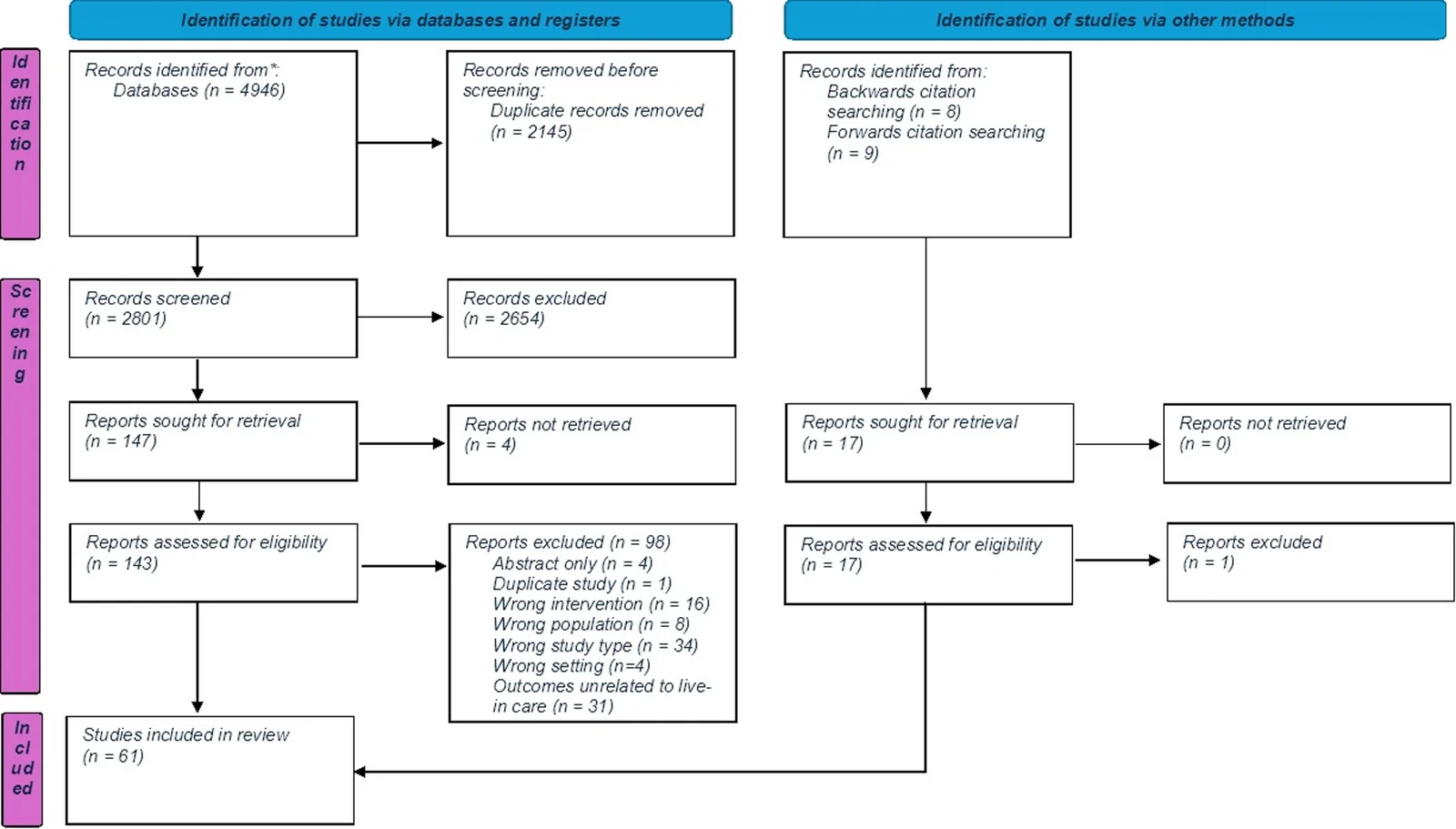
PRISMA flow diagram showing flow of studies through the review

After completion of the database searches, 4946 records were identified and deduplicated. 2801 abstracts were screened, resulting in 160 full text documents being assessed for eligibility. In total, 61 studies were included in the scoping review (see Fig. 1, PRISMA flow diagram).

### Study characteristics

Israel featured in by far the greatest number of studies (*n* = 23) followed by Taiwan (*n* = 7). Overall, studies were conducted in Israel (*n* = 23) [9–31], UK (*n* = 5) [32–36], Germany (*n* = 5) [37–41], Canada (*n* = 2) [42, 43], Italy (*n* = 1) [44], Switzerland (*n* = 5) [45–49], Taiwan (*n* = 7) [50–56], Belgium (*n* = 1) [57], Austria (*n* = 2) [58, 59], USA (*n* = 1) [60], Malta (*n* = 1) [61], Singapore (*n* = 1) [62], Hong Kong (*n* = 1) [63], Spain (*n* = 1) [64], Netherlands (*n* = 2) [65, 66] and Kuwait (*n* = 1) [67]. Two studies were carried out in two different countries: one in Germany/Netherlands [68] and the other in Germany/Israel [69]. Studies were published between 2007 and 2025, with most studies published post 2018 (*n* = 42).

The majority of studies were qualitative in nature (*n* = 39) [9–12, 15, 16, 29, 32–42, 44–47, 49, 51–62, 65, 66, 68, 69], with 14 quantitative studies [17, 18, 20–22, 24–28, 30, 64, 70, 71] and eight employing mixed methods [13, 14, 19, 31, 43, 48, 50, 63]. The quantitative studies were all cross-sectional in design and were conducted using surveys. Qualitative studies used interviews or focus groups except for one study [41] which carried out an analysis of blogposts.

Table 1 gives an overview of the included studies.

**Table 1:** Characteristics of included studies

First author, date	Country	Study design	Population	Objective	Topics addressed
Ahlberg 2022	UK	Qualitative	14 live-in carers	To understand better the risks and drivers of vulnerability to modern slavery and severe forms of labour exploitation	• Migration • Exploitation of workers • Employment status/contracts • Nature of work • Blurring of boundaries
Arieli 2025	Israel	Qualitative	37 live-in carers	To explore how live-in migrant care workers experience and cope with their care recipients’ end-of-life and death phases	• Mental health (live-in carers) • Migration • End of life care • Carer/older person relationships
Arieli 2024	Israel	Qualitative	35 family members	To explore family members’ experiences in employing live-in care workers	• Motivations for choosing live-in care • Setting of boundaries • Trust issues
Ayalon 2009a	Israel	Qualitative	29 live-in carers 22 family members	To investigate emotional and instrumental transfer of care from family member to live-in carer	• Motivations for choosing live-in care • Carer/older person relationships • Carer/family members relationships
Ayalon 2009b	Israel	Qualitative	7 older people 23 family members	To investigate the challenges associated with foreign carers from perspectives of older people and family members	• Care quality • Trust issues • Conflict
Ayalon 2009c	Israel	Mixed methods	245 live-in carers 22 family members 31 social workers	To evaluate the working conditions and work-related abuse of Filipino care workers in Israel	• Nature of work • Mental health (live-in carers) • Exploitation
Ayalon 2011	Israel	Mixed methods	148 live-in carers 148 family members 90 older adults	To evaluate satisfaction with migrant live-in care from all perspectives	• Care quality • Job satisfaction • Mental health/wellbeing (all)
Ayalon 2015	Israel	Qualitative	20 live-in carers 16 family members 17 older adults	To understand the roles of live-in carers and examine difficulties	• Carer/family member relationships • Carer/professional staff relationships • Nature of work
Ayalon 2025	Israel	Qualitative	21 live-in carers 19 family members	To address perspectives of family members and live-in carers on ‘behaviour problems’ in people with dementia.	• Caring for a person with dementia • Carer/older person relationships • Carer/family members relationships • Cultural differences
Basnyat 2017	Singapore	Qualitative	20 family members	To investigate primary caregivers’ relationship with hired support and its impact on coping with caregiver burden	• Caring for a person with dementia • Carer/family member relationships • Nature of work
Boccagni 2018	Italy	Qualitative	165 live-in carers	To explore how migrant workers negotiate a domestic space, their interactions with care recipients and working/living conditions	• Migration • Setting of boundaries • Carer/family member relationships • Carer/older person relationships • Blurring of boundaries • Living conditions • Nature of work
Bourgeault 2010	Canada	Qualitative	19 live-in carers	To understand the impact that working under the Canadian live-in care program has on carers working and living experiences and relationships with clients	• Carer/older person relationships • Carer/family member relationships • Nature of work • Migration • Employment status/contracts • Living condition • Training
Chau 2019	Switzerland	Qualitative	13 live-in carers 20 agency reps	To explore the politics of mobility in organisation of live-in care arrangements	• Packages of care • Employment status/contracts • Cost/funding
Chau 2018	Switzerland	Qualitative	12 live-in carers 20 agency reps 2 workers reps	To investigate the difficulties faced by migrant live-in carers in Basel	• Migration • Packages of care • Living conditions • Nature of work • Job satisfaction
Chowdhury 2012	Canada	Mixed methods	14 live-in carers	To explore characteristics of live-in carers under Canadian programme and explore job satisfaction	• Job satisfaction • Training • Carer/older person relationships • Employment status/contracts
Cohen-Mansfield 2017	Israel	Quantitative	111 live-in carers	To examine the living and working conditions of live-in carers	• Living conditions • Nature of work
Cohen-Mansfield 2019	Israel	Quantitative	98 live-in carers 61 older people 111 family members	Social engagement provided by live-in carers for frail older adults	• Nature of work • Carer/older person relationships
Cohen-Mansfield 2021	Israel	Quantitative	116 live-in carers	To clarify how work characteristics affect migrant live-in care workers burden/burnout and job satisfaction	• Job satisfaction • Carer/older person relationships • Mental health/wellbeing (carers) • Caring for a person with dementia
Cohen-Mansfield 2022	Israel	Quantitative	115 live-in carers 72 older people 117 family members	To examine the association between employment related characteristics and the quality of care	• Care quality • Carer/older person relationships • Carer/family member relationships • Nature of work • Cultural differences
Cohen-Mansfield 2023	Israel	Mixed methods	116 live-in carers 117 family members	To understand how live-in carers and family members perceive nature of their relationship	• Carer/family member relationships • Carer/older person relationships • Trust issues • Nature of work • Conflict • Cultural differences
Cohen-Mansfield 2018	Israel	Quantitative	72 older people 117 family members	To establish the components of quality of care as provided by live-in carers	• Care quality
Gerhards 2022	Germany	Qualitative	182 family members	To explore moral conflicts that arise with live-care arrangements for people with dementia	• Conflict • Blurring of boundaries • Caring for person with dementia • Cultural differences • Employment status/contracts • Nature of work
Green 2018	Israel	Quantitative	338 live-in carers	To assess working conditions and prevalence of abuse and exploitation among live-in carers	• Employment status/contracts • Exploitation of workers • Living conditions • Nature of work
Grenz 2024	Germany	Qualitative	15 family members	To learn about the experiences of family members with live-in care models for people with dementia	• Employment status/contract • Packages of care • Nature of work • Caring for person with dementia • Conflicts • Care quality
Gum 2022	Israel	Quantitative	6 live-in carers	To test an intervention to increase valued activities for carers and older people	• Caring for a person with dementia • Mental health/wellbeing (carer) • Mental health/wellbeing (older person) • Carer/older person relationships
Haslinger-Baumann 2019	Austria	Qualitative	1 older person 8 live-in carers 14 family members 9 registered nurses	To investigate perceptions of 24 h care quality	• Care quality • Blurring of boundaries • Training • Carer/older person relationships
Heng 2019	Netherlands	Qualitative	11 live-in carers	To explore the caregiving experiences and coping strategies of foreign domestic workers caring for frail older people	• Mental health/wellbeing (carers) • Coping strategies • Support/protection for carers • Training
Hoens 2023	Belgium	Qualitative	6 live-in carers 9 older people/family members	To explore experiences of older people and their families in hiring live-in care	• Packages of care • Cost/funding • Motivations for choosing live-in care • Carer/older person relationships • Care quality • Nature of work • Employment status/contracts • Training
Hopfgartner 2022	Austria	Qualitative	7 live-in carers	To examine live-in carers perceptions of job	• Employment status/contracts • Training • Nature of work • Coping strategies • Exploitation of workers
Horn 2019	Germany/ Netherlands	Qualitative	24 family members	To explore family motivations for using live-in carer	• Motivations for choosing live-in care • Employment status/contracts
Hsu 2025	Taiwan	Mixed methods	270 older people 268 family members 270 live-in carers	To examine satisfaction and relationships between the triads	• Carer/older person relationships • Carer/family members relationships • Job satisfaction • Care quality • Cultural differences
Huey Shy 2021	Switzerland	Qualitative	13 live-in carers 20 agency reps	To explore activities of private employment agencies that recruit live-in carers	• Package of care • Employment status/contracts
Hussein 2020	UK	Qualitative	4 live-in carers	To highlight the motivations, experience and future plans of care workers in England	• Employment status/contracts • Nature of work • Training
Hussein 2024a	UK	Qualitative	26 live-in carers	Experiences of live-in carers following Brexit	• Migration • Employment status/contracts • Exploitation
Hussein 2024b	UK	Qualitative	8 live-in carers	To investigate the interplay between migration and social care systems on carers wellbeing	• Migration • Mental health/wellbeing (carers)
Hung 2024	Hong Kong	Mixed methods	288 older People	To explore how quality of relationships affects loneliness in older people	• Carer/older person relationships • Mental health/wellbeing (older person) • Cultural differences
Iecovich 2007	Israel	Quantitative	39 older people	To compare satisfaction between live-in and live-out carers	• Cultural differences • Carer/older person relationships • Care quality • Job satisfaction
Iecovich 2011	Israel	Quantitative	335 older people 335 live-in carers	To investigate job satisfaction and quality of relationship between live-in carer and older person	• Carer/older person relationships • Job satisfaction
Iecovich 2016	Israel	Quantitative	279 older people 279 family members 279 live-in carers	To examine relationships between older people, family members and live-in carers	• Carer/older person relationships • Carer/family member relationships • Mental health/wellbeing (older person)
Kriegsmann-Rabe 2023	Germany	Qualitative	15 live-in carers	To understand factors that promote carers wellbeing and resilience	• Mental health/wellbeing (live-in carers) • Packages of care • Employment status/contracts • Exploitation of workers
Liang 2023	Taiwan	Qualitative	29 family members	To examine why families hire live-in carers and the the qualitative consequences	• Motivations for choosing Live-in care
Lin 2013	Taiwan	Qualitative	43 live-in carers 23 family members 7 older people	To understand how families and live-in carers negotiate care work and kinship ties	• Motivations for choosing live-in care • Cost/funding • Training
Lin 2012	Taiwan	Qualitative	20 live-in carers	To examine how long-term co-residence of live-in carers affects families	• Carer/family member relationships • Carer/older person relationships • Blurring of boundaries
Munkejord 2021a	Taiwan	Qualitative	10 live-in carers 10 family members	To explore the phenomenon of live-in care	• Exploitation of workers • Nature of work • Carer/older person relationships • Caring for a person with dementia
Munkejord 2021b	Taiwan	Qualitative	13 live-in carers	To explore the coping strategies of live-in carers	• Mental health/wellbeing (live-in carers) • Nature of work • Carer/older person relationships • Coping strategies
Ness 2023	Taiwan	Qualitative	10 live-in carers 11 family members	Exploring the transition from family carer to employer of live-in carer	• Motivations for choosing live-in care • Mental health/wellbeing (family members) • Care quality • Trust issues • Cultural differences
Petry 2016	Switzerland	Mixed methods	14 older people 21 family members	To investigate families’ experiences with live-in carers	• Motivations for choosing live-in care • Care quality • Packages of care
Porat 2010	Israel	Quantitative	100 live-in carers 100 older people	To explore relationships between live-in carers and older people	• Carer/older person relationships
Regev-Messalem 2020	Israel	Qualitative	52 family members	To understand how family members evaluate live-in care in terms of monetary value	• Cost/funding • Packages of care • Carer/family members relationships • Conflict
Rezola-Pardue 2025	Spain	Quantitative	43 live-in carers	To compare mental and physical health between live-in and live-out carers	• Mental health/wellbeing (carers) • Physical health (carers)
Riley 2016	USA	Qualitative	32 live-in carers	To document the sleep patterns and outcomes for live-in carers	• Nature of work • Packages of care • Living conditions
Schilgen 2020	Germany	Qualitative	9 live-in carers	To explore situation of live-in carers with focus on workplace stressors and resources	• Nature of work • Mental health/wellbeing (carers) • Conflict • Blurring of boundaries • Living arrangements • Coping strategies
Shah 2012	Kuwait	Quantitative	808 care recipients	To examine health status of older people cared for by domestic workers	• Mental health/wellbeing (older people) • Physical health (older people)
Shinan-Altman 2019	Israel	Quantitative	338 live-in carers	To identify factors that contribute to perceived control	• Carer/older person relationships • Carer/family member relationships • Job satisfaction • Mental health/wellbeing (carers)
Teshuva 2019	Israel	Mixed methods	116 live-in carers 73 older people	To explore the relationships between live-in carers and older people	• Carer/older person relationships • Care quality • Job satisfaction • Cultural differences
Ulitsa 2025	Israel/Germany	Qualitative	24 commissioners	To understand vulnerabilities in dementia care with live-in carers	• Caring for a person with dementia • Conflict • Mental health/wellbeing (carers) • Carer/older person relationships • Cultural differences • Nature of work
Van Bochove 2020	Netherlands	Qualitative	9 live-in carers 8 professionals 3 family members	To understand how care networks shape experience of live-in carers	• Carer/family member relationships • Living conditions • Conflict
Van Holten 2019	Switzerland	Qualitative	11 family members	To examine the notion of familiarity of live-in care settings for older people	• Motivations for choosing live-in care • Setting of boundaries • Conflict • Cultural differences
Vandrevala 2022	UK	Qualitative	21 live-in carers	To examine challenges and mitigations faced by live-in carers	• Nature of work • Carer/older person relationships • Carer/family members relationships • Mental health/wellbeing (carers) • Coping strategies
Vassallo 2020	Malta	Qualitative	8 live-in carers	To explore the reality of live-in carer	• Migration • Nature of work • Carer/older person relationships • Support/protection for carers • Blurring of boundaries
Wustner 2024	Germany	Qualitative	Unknown (blog posts)	To explore power held by live in carers in Germany	• Exploitation of workers • Cost/funding • Coping strategies • Setting of boundaries

### Population

Across the studies, 3391 interviews were carried out with live-in care workers, 1720 interviews or surveys were carried out with care recipients and 1709 with family members. Given the number of related studies (i.e. by the same author or team of authors), it is highly likely that the same participants were involved in interviews reported across multiple studies. Participants in other studies included social workers (31 interviews), registered nurses and other professionals (16 interviews), agency representatives (60 interviews), and live-in dementia care ‘experts’ including service providers, social workers and government representatives [69] (24 interviews).

Characteristics and co-morbidities of the care recipients were rarely reported, and usually in vague terms for example 86.3% were severely disabled in terms of activities of daily living [26], 7% suffered memory impairment [48] or 4.8% were unable to leave their beds [71]. Where studies reported on gender, most care recipients were female.

In all studies the live-in carers were wholly or predominantly migrant workers/workers from overseas. Although nationalities of live-in carers were rarely reported, countries of origin included The Philippines, Nepal, India, Sri Lanka, Moldova, Ukraine, Uzbekistan, Ecuador, Peru, Poland, Romania, Indonesia, Myanmar, Bulgaria, Iran, Slovakia, Hungary, Vietnam, Zimbabwe, South Africa, Thailand and Kosovo.

### Live-in care for people with dementia

Only seven studies focused on live-in care for people with dementia [16, 17, 24, 37, 38, 62, 69] and none of these included or interviewed the people with dementia. One study [10] reported that 90% of care recipients had dementia but only interviewed family members. All studies focused on dementia were conducted in Germany or Israel apart from one [62], which was conducted in Singapore. Although several other studies reported that dementia was a common comorbidity [20, 39, 48–50, 55, 64, 65], none provided percentages.

Table 2 gives an overview of the 7 studies that focus on live-in care for people with dementia.

**Table 2:** Studies focusing on dementia care

Study	Country	Subject	Focus of study	Findings
Ayalon 2025	Israel	21 live-in carers 17 family members	Challenges of caring for a person with dementia	Similar perspectives between 2 groups concerning challenges and management. Family members have role as mediators.
Basnyat 2017	Singapore	20 family members	Relationships with live-in carer	Live-in carers help to reduce burden and worry for family members
Cohen-Mansfield 2021	Israel	112 live-in carers (33 caring for person with dementia)	Job satisfaction and burnout in carers	Fostering better relationships and improving employment conditions could reduce burnout and improve satisfaction
Gerhards 2022	Germany	182 family members	Expectations of relatives towards live-in carers	Expectations of relatives are diverse and often disappointed
Grenz 2024	Germany	15 family members	Conceptualisations of good care and conflicts	Highlights importance of recognising needs of the ‘triad’ (person with dementia, family member and live-in carer).
Gum 2022	Israel	6 live-in carers	Behavioural activation for carer and care recipient	Joint activities increased leading to better satisfaction
Ulitsa 2025	Germany/Israel	24 ‘experts’ in providing at home dementia care	Vulnerabilities in live-in care systems	Vulnerabilities are deeply entangled within care triads. Addressing these is essential to improve care quality and wellbeing of all.

### Themes

The themes described below (motivations for choosing live-in care, recruitment, packages of care, contracts, training, nature of work, satisfaction with care and relationships) were generated based on findings from the included studies identified during data extraction, the relevance to the research questions and suggestions made by the lay advisory group. Topics raised by the Lay Advisory Group as being of interest that are discussed below were quality of care, cultural differences, the arrangement of care packages, the nature of the role and interactions/relationships. The group (which was UK based) was also interested in the impact of Brexit on the live-in care workforce, but this was beyond the scope of this review. The findings are described first in relation to live-in care for all older populations, and then with a focus on dementia care. Themes are presented sequentially i.e. beginning with initial motivations of family members to seek live-in care, rather than by the amount of coverage within the studies.

#### Motivations for choosing live-in care

Twelve studies detailed motivations behind families’ choice of live-in care for their relatives [10, 12, 13, 38, 48, 49, 52, 56, 57, 62, 68, 69].

The decision to employ a live-in carer was usually made by family members due to prolonged deterioration in the health or functional ability of the care recipient, or a sudden incident e.g. a stroke [10, 48, 56, 68]. In some cases, external crises (e.g. death of a spouse ) also contributed to the decision [68].

Live-in care was often considered the only viable option as other options for 24-hour care were too costly [11, 68] or considered insufficient and restricted by budget constraints and fragmented systems [57, 68]. Furthermore, there was a negative perception about nursing homes [11, 48, 57, 68] and home care services were considered inflexible, meaning care recipients and family members had less control over their lives e.g. because they had to meet appointment times or be responsive to issues that arose [56, 57, 68].

Several studies describe strong beliefs across all populations that ageing in place in a familiar environment is the best option [49, 52, 57], and for care recipients to be near to visiting friends and neighbours in the community [49]. Promises might be made between spouses or family members that care would be provided at home [49]. Additionally, cultural expectations or beliefs meant that caregivers rejected removing family members from the home [62]. In another study, partners wanted to spend as much time as possible with dependent spouses and it was hoped that live-in care would provide company for the care recipient [57].

#### Motivations: dementia care

Those caring for family members with dementia found caring to be a full-time job, sacrificing their own personal lives which resulted in loneliness or mental stress [62]. Studies described family members struggling with work commitments as well as feeling overburdened by the demands of caring [10, 56, 57], or living too far away to help with caring [48], or of struggling to share out caring responsibilities amongst siblings [56]. Family members felt unqualified or incapable of providing suitable care, which was particularly true where care needs were complex [10, 48], or where carers felt it was dangerous to leave family members unattended [10]. Often family carers were older adults themselves [62] and at risk of age-related health conditions.

Family members of people living with dementia appeared to have similar motivations for considering live-in care to those of family members of people with other conditions. In some studies, care recipients themselves were reluctant to contemplate accepting live-in care, because they felt this increased awareness of their vulnerability exacerbated dependency and removed autonomy [10, 48, 69] and they feared loss of personal dignity. Furthermore, care recipients feared that the employment of a live-in carer would mean less contact with their relatives [69].

#### Recruitment

Recruitment of live-in care workers in all countries typically happened via private agencies [46, 48, 57, 60]. Other methods of recruitment were via placement (matching) agents, carer websites or privately, whereby the family or the care recipient became the direct employer. Using an agency had the advantage of providing swift access to live-in care, often in as little as 6–10 days with swift replacements provided during carer absences [45, 48]. Agencies also usually dealt with arrivals, wages, taxation, contracts and permits for migrant workers. Recruitment was no different for live-in care work when the recipient had dementia.

#### Packages of care

Care packages were intrinsically linked to residency permits for migrant workers i.e. how long workers were permitted to stay in the country. Contracts were often short-term, meaning that live-in carers returned to their families in home countries, operating on a rotation basis with other carers [38, 46]. Work patterns varied across countries and agencies. A typical pattern for a UK live-in carer was 8–10 weeks working followed by 2 weeks off [33] and, where described, carers were self-employed or working with a zero-hour contract [32]. Agency care packages sometimes varied according to needs of the care recipient [45].

#### Packages of care: dementia care

Where the care recipient had dementia, continuity of care (constant provision of live-in care) was cited as particularly important for families [38, 48].

#### Contracts

Contracts and wages varied according to legal requirements in different countries. However, contracts were often vague [32, 57] e.g. payment for bedroom, food, transport and cosmetics, such as body lotions for the care recipient, fell into grey areas [10, 43]. Occasionally contracts were not even provided [70] or carers were not paid on time [42]. Those recruited privately were less likely to have contracts than those recruited via agencies [60].

Studies found that far longer hours were worked than stipulated [43, 61]. Carers could be paid for working 40–48 h a week but were often not paid for the many more hours they were on call (68) and several studies reported carers being on 24-hour call, even during breaks [32, 33, 42, 46, 57, 60].

Studies reported that despite fair treatment of live-in carers being important to care recipients and family members, they struggled to meet wage and hour regulations required by law, particularly in EU countries [38, 48, 68]. Even where live-in care work was regulated, self-employment and zero-hours contracts rendered employment laws inapplicable [32, 59]. Life for many live-in carers was insecure with unexpected job terminations if they complained about long hours or nature of the job [32, 36, 41].

#### Contracts: dementia care

For live-in workers caring for people with dementia, aspects of their job e.g. working hours could change as the dementia worsened with no renegotiation of contract [59].

#### Training

Eight studies [33, 42, 43, 52, 57–59, 65] describe training of live-in carers in any detail. Although live-in carers were educated to a range of levels, no studies described any formal system of live-in care training or qualification, unless mandated by overseas work.

##### Government requirements

The Live-in Caregiver Program in Canada required at least 6 months of full-time training in a nursing related field, or 1 year of experience as a caregiver [43]. Other than this, no studies indicated that placement agencies demanded specialist knowledge.

Informal training for live-in carers was sometimes provided by agencies [57, 65] although this was often described as inadequate. Sometimes agencies charged carers extra for training and if a carer changed agency, new training had to be undertaken [33, 42]. Families provided training themselves, even in medical procedures such as administering insulin and measuring blood pressure [58, 65] or carers were expected to learn on the job [33, 57–59]. The lack of medical training meant live-in carers encountered stressful situations e.g. trying to resuscitate a care recipient or having to make decisions about whether to call relatives or an ambulance [9]. Medical procedures such as blood pressure monitoring or providing insulin injections were ambiguously situated between basic care and medical care [58]. Even if live-in carers were not mandated to carry out medical procedures, this was not always understood [58].

Training, if provided, usually consisted of basic nursing care such as changing dressings or carrying out household chores. If the carers were from culturally different places, training related to culture was provided e.g. food preparation, bed making and mealtime arrangements [43, 53].

#### Training: dementia care

None of the studies described specific training for live-in carers with regards to dementia although Basnyat [62] refers to a government scheme in Singapore whereby family caregivers can pay for live-in care workers to attend training programs.

#### Nature of work

Many (*n* = 24) studies [13, 15, 18–21, 32, 33, 36–38, 40, 42, 44, 46, 54, 55, 57, 59–62, 69, 70] detail the nature of the work carried out by live-in carers. Typical basic care tasks described were administering medication, helping with food and mealtimes, bathing, grooming, encouraging exercise, changing incontinence pads, taking blood pressure, measuring blood sugar or other vitals and basic housekeeping [13, 37, 55, 60]. In addition, live-in carers provided emotional care and companionship [13, 15, 20, 42].

However, as with working hours, the exact nature of the job was often poorly defined. Carers were expected to carry out tasks beyond caring such as gardening, or more exploitative tasks such as driving family members around, chopping wood or preparing a dinner party for guests [32, 40, 42, 57]. In some families, the live-in care workers described being viewed by family members as servants [40, 54]. Several studies described poor living conditions for carers, including freezing or dirty rooms, no private room at all [40, 70] or not having access to enough food [13, 32, 42]. Insufficiently defined responsibilities meant there was often a blurred separation between work and leisure time [32, 37, 40, 61].

#### Nature of work: dementia care

Caring for an older person with dementia was associated with care worker burden and a strong indicator of the care workers’ intention to leave a job [17, 62]. Several studies described live-in carers lacking sleep, which was accentuated if the care recipient was living with dementia [36, 40, 43, 55, 60, 69].

#### Satisfaction with care

Twelve studies investigated factors affecting satisfaction with care [12, 14, 18, 22, 25, 31, 38, 48, 50, 56–58].

Factors that were important to the care recipients were care workers competencies, their recognition of physical care needs, satisfaction with dietary arrangements, ability of workers to interact and understand the care recipient, and their flexibility in responding to requests [22, 31, 48, 50, 58]. A study from Austria [58] identified several criteria relating to live-in care quality, including fulfilling basic care duties (washing, personal hygiene, cooking, cleaning, outings, entertainment and having conversations), training, and individualised (person-centred) care. Care recipients also wanted care workers to be clean, hard-working, honest, organised, compassionate, happy and punctual, with skills in cookery [22, 31, 48, 50, 58].

Several studies gave examples of poor care. One study from Israel [12] described issues with invasions of privacy, changes to routines, trust, emotional or financial abuse or neglect. Yet in many cases the live-in care arrangement was positive for all. High satisfaction with care was described in studies as correlating with live-in carer job satisfaction [14], good relationships between the care worker and the clients (care recipients and family), and satisfactory conditions of employment [18]. Quality of care was also deemed good when carers took pride in giving the best care and made efforts to foster joint enjoyment e.g. through dancing or baking [31]. 

#### Satisfaction: dementia care

Difficulties were exacerbated with dementia care if the care recipient was fearful of their care worker or if family members believed these fears were a symptom of the dementia and therefore deemed them unfounded [12].

#### Cultural differences

Descriptions about cultural differences between live-in care worker and care recipients arose in 11 studies [16, 18, 19, 25, 31, 37, 49, 50, 56, 63, 69] for example in relation to meal preparation [49, 50], where food preferences and religious dietary restrictions applied. Cultural differences between care workers and care recipients resulted in a diverse range of relationships across the studies, from workers being treated like family members [31] to workers receiving racial or discriminatory abuse [69]. Beliefs shaped relationships [63] and cultural differences were cited by study participants as a reason for weaker relationships [19]. Where cultural differences existed, live-in care workers sometimes found relationships challenging, causing them stress, isolation and burnout [69]. Where care workers were treated as family members, perceived class and ethnic differences diminished [53].

#### Cultural differences: dementia care

In a study carried out in both Germany and Israel, language barriers and cultural differences between the care worker and the care recipient with dementia was deemed the source of misunderstandings and conflict [69]. A study from Israel [16] suggested that cultural differences might contribute to fear or agitation in the care recipient with dementia. Family members in this study highlighted their role in having to mediate care givers’ knowledge about dementia as well as familial relationships, customs and holidays in Israeli society to ensure sufficient care was provided.

#### Relationships

32 studies investigated aspects of the relationships between live-in carers, care recipients and family members [9, 11, 15–21, 24–31, 36, 42–44, 50, 53–55, 57, 58, 61–63, 66, 69]. Four studies discussed trust [9, 10, 12, 19, 56] and one examined end-of-life care relationships [10].

Relationships were strengthened through mutual respect. It was crucial that the care worker did not change household arrangements without consultation, and that older people were willing to adapt to their new living arrangements and be prepared to hand over responsibilities to their carer [31, 48, 63]. Where care workers were treated with respect, positive relationships were described [26, 31, 42, 53, 57] and fostered when carers were able to treat the home like their own and could go about basic tasks e.g. going to the fridge for food without feeling uncomfortable [36]. Being supplied with a private room with adequate heating, a comfortable bed and use of a washing machine, TV, radio and computer were important factors for live-in care workers [21, 44]. Having their own key and being able to meet or invite friends or family members over was also deemed important [44]. Autonomy for care workers was also associated with more positive relationships [30].

Communication was key to a good relationship [58, 63]. For care workers, being listened to, and being open and honest were important and care recipients valued interesting conversations [31]. Care recipients and live-in care workers described becoming close, like friends [31, 57] or even family members [28, 31, 53, 57] with care workers describing care recipients as being like a parent or grandparent to them [9, 11].

Difficulties with close bonds did arise. For example, devoted care workers felt deep grief at the end of life [9]. Furthermore, care workers who were regarded more like family members ran the risk of being taken for granted [53, 63], with family members requesting additional unpaid labour without feeling guilty [53]. Emotional attachment also restricted care workers from seeking more autonomy [44]. Relationships between the care workers and family members was also crucial for successful live-in care arrangements. If the relationship with care recipient was positive, often the relationship with family members was also positive [19].

#### Relationships: dementia care

Good relationships between care workers and older people with dementia developed when care workers engaged intensively with them, understanding their needs and preferences with shared activities such as watching TV together, playing boardgames, meals, walks, trips to cafes, potting plants and dancing [24, 38, 48]. However, fears that a trusted care worker would leave were exacerbated when the care recipient had dementia [69].

Misunderstandings, exacerbated where the care recipient had dementia, sometimes led to conflict [9, 31, 58, 69]. However, displaying caring behaviour could compensate in these situations [25, 28, 31].

**Fig. 2:**
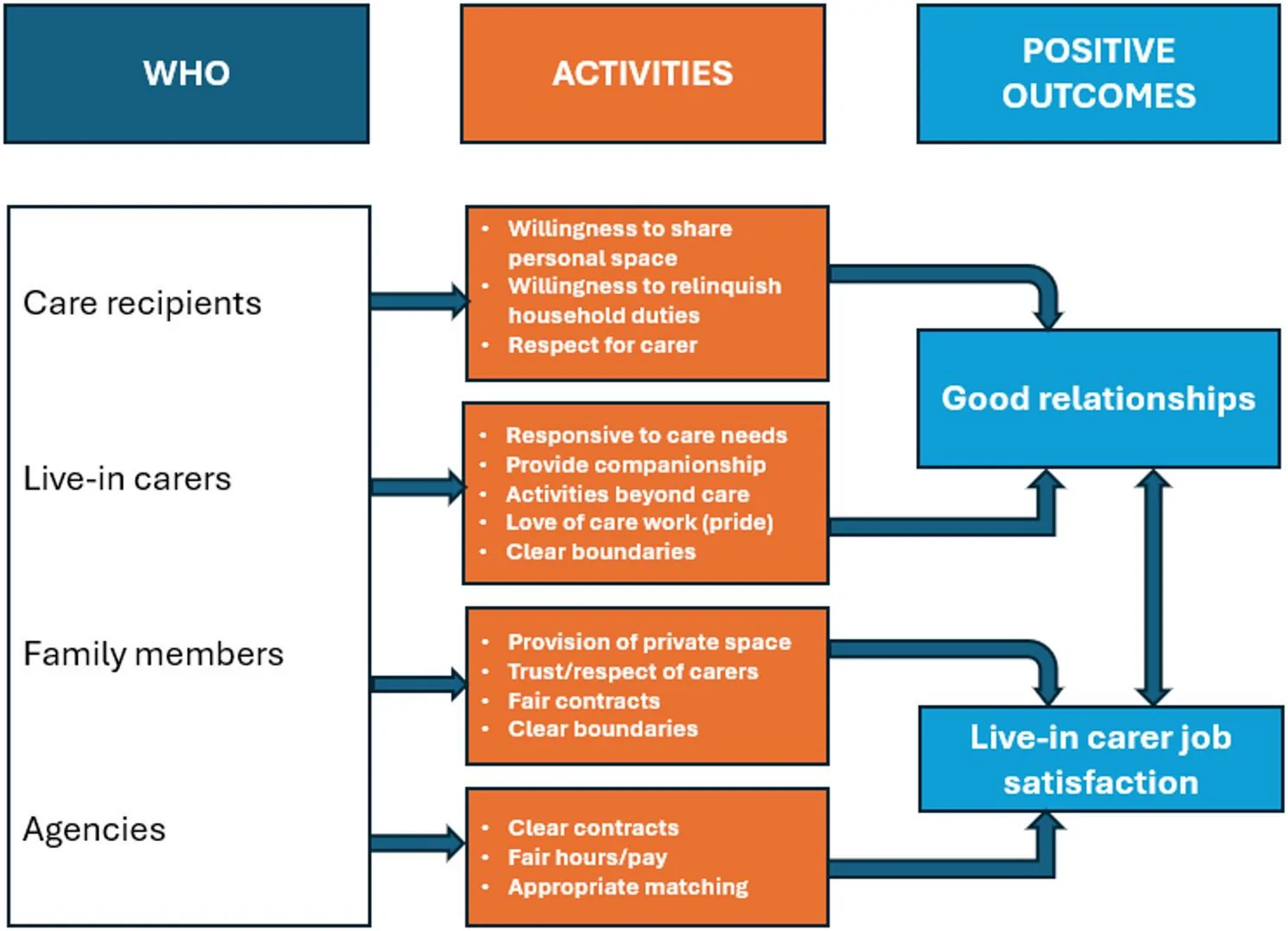
Factors impacting on relationships and live-in carer satisfaction

Figure 2 shows the factors impacting on relationships and live-in carer satisfaction as reported in the studies.

## Discussion

This scoping review aimed to describe the volume, range and nature of evidence on live-in care for older adults and investigate the evidence on whether live-in care supports ageing in place for older adults with dementia.

A total of 61 studies were identified that described the experience or effectiveness of live-in care, the majority of which were qualitative in design. Most studies did not report the proportion of care recipients with dementia and only seven focused on older care recipients with dementia. Studies were usually conducted in high-income countries where live-in care was typically provided by migrant workers. Regardless of country, studies described similar motivations for utilising live-in care. Populations across the globe are ageing and demands are the same with fragmented systems, a mistrust of residential care, prohibitively expensive care options, relatives/family carers unable to cope with caring duties as well as work, and a strong belief that ageing in place and at home is best [1–5]. Much of the literature highlights the precarious nature of this work and the evidence base on live-in care for older adults is heavily skewed toward the perspectives of live-in care workers. Although not a focus of this review, exploitation of the workforce, as found in previous work [6] was often described.

The dominance of the carer (formal or informal) perspective reflects a broader trend in care research. Previous work has identified that the voices of people with dementia are frequently absent from the evidence base (e.g. Fletcher, 2020, Halonen, et al., 2025) [72, 73]. This review also highlights a substantial gap in our understanding of live-in care from the standpoint of older adults including people with dementia. Addressing this gap will require future research to create inclusive environments in which diverse voices can be heard. However, this takes time, skill and careful planning, which may not always be available within academic research teams [74]. Home-based fieldwork also presents distinctive practical and ethical challenges for both researchers and participants, including physical and emotional risks associated with lone working and the potential for encountering domestic abuse [75]. Future research on live-in care should therefore use creative methods to elicit information and involve multiple perspectives, to develop a more rounded understanding of the arrangement. In this way, we might move towards understanding dementia as a shared experience [76].

Live-in care was more successful where the carer had a contract, where there was an understanding of job requirements and clearly defined boundaries, for example, clear guidance with regards to food purchases, travel costs and other expenses and a good ‘match’ between the customer (i.e. the care recipient and family members) and the live-in carer. Carers required a private, comfortable space, access to basic appliances, fair pay for hours worked, dedicated time off and fixed boundaries. Care recipients required good quality of care from carers who were responsive to their needs. The findings of this review therefore support previous studies calling for clearer contracts or a code of ethics endorsed by both employers and live-in carers [32, 61].

Studies found that where activities fostered good relationships, there was higher satisfaction with the care provided. This insight resonates with the vast body of evidence from care home research that person-centred and relational care are fundamentally components of a quality service [77]. Given the strength that good communication and relationships have on satisfaction with live-in care, and caregiver burden and job satisfaction, agencies could consider an emphasis on matching carers and clients with similar qualities, backgrounds and beliefs. Considered matching could enable stable, continuous relationships allowing the live-in carer to develop a deep understanding of the individual’s needs, habits, and preferences over time. Of course, matching is not always possible and other solutions are available. For example, research shows that providing culturally competent care is key to reducing disparities and is more scalable [78]. The key to facilitating ‘ageing in place’ for older people, including those living with dementia is to ensure that care preferences match the care arrangements [79].

One implication of this study is that ageing in place is valued not only by policymakers but also by families and spouses. Such commitments often stem from negative perceptions of residential care: in countries including Belgium, Germany, Switzerland and Israel, care homes are frequently viewed as offering substandard care, or families simply do not trust this form of provision [11, 48, 57, 68]. More broadly, the familiar expression ‘there’s no place like home’ reflects a widely held belief that one’s own environment provides comfort and security, both physical and psychological. In this sense, ageing in place emerges as a cornerstone not only of family relationships but also of contemporary health and social care policy.

Future research needs to explore live-in care from multiple perspectives including people living with dementia. There is no ‘right’ perspective or single truth about live-in care (or any other form of care). Therefore, it is important to elicit information from all those involved in the arranging, delivery, and receipt of live-in care, including people with dementia. For this reason, it will be important for future work to take a more creative and flexible approach to data collection. Most of the qualitative studies included in this review relied on interviews, which may not be suitable for a person with dementia, given the method requires recall and language skills. Future work could consider employing other methods such as observation and diaries to create more opportunities for participation. Given the variety of perspectives that are relevant to understanding live-in care and need for robust evidence to inform policy and practice, future research must continue to adopt a mixed-methods design. Only 13 per cent of the studies in this review used a mixed method design, yet the inclusion of both qualitative and quantitative data can help to triangulate data and provide rich sources of analysis [80]. As such, there is a pressing need for studies to use mixed method designs to ensure a full understanding of live-in care is gained.

Studies within this scoping review provided little insight into how care packages were arranged and delivered by commissioners or agencies, how live-in carers were recruited, what barriers or problems were encountered to the provision of live-in care, and whether training for live-in carers was sufficient or effective or even provided. Future research might also seek to address these gaps.

### Strengths and limitations

This scoping review is the first to examine live-in care for older adults with a specific focus on dementia. It followed recognised guidelines and included a rigorous literature search and study selection by two independent researchers. The comprehensive literature search found far more studies than previous reviews and covered a broader geographical range. Our inclusion of non-English language papers meant that we were able to examine live-in care arrangements in European countries such as Germany and Switzerland whereas previous reviews had reported an absence of literature from Europe.

A second strength was the contribution of the lay advisory group, which suggested terms for inclusion in the literature search and identified which factors of live-in care were most important to them, enabling us to shape the findings accordingly.

In line with guidance on the conduct of scoping reviews, critical appraisal of the included studies was not carried out. However, the aim of this review was not to establish the effectiveness of live-in care but to gather studies investigating live-in care, with a view to aiding care commissioners, agents, carers and care recipients in making decisions about appropriate live-in care solutions that facilitate ageing in place.

## Conclusions

Live-in care provides the potential to allow older people and those living with dementia to ‘age in place’. Existing research finds that this work is provided predominantly by migrant workers who are over-burdened and open to exploitation. The studies identified were mostly qualitative in nature and focused on experiences of live-in care workers and the clients (older people, people with dementia and their family members).

A consistent finding was that good relationships between care workers and the care recipients were key to satisfaction both for the care worker and the care recipient. Given this, choice of carer should be based on factors that promote positive relationships e.g. similarities in beliefs and culture. Studies consistently concluded that boundaries should be clearly defined, for example in terms of food purchases and travel expenses. Contracts that recognise different levels of care along with an agreed code of ethics for both employers and live-in care workers would help facilitate good relationships. Specialised dementia care training for live-in care workers may also facilitate improved experience for both carer and care recipient. More research into these specific areas could provide implications for practice.

Live-in care for people with dementia can be successful although factors affecting its success (good communication, reciprocal trust, increased care burden) may influence its outcomes.

## Supplementary Information

Supplementary Material 1.

## Data Availability

All datasets used or analysed in this scoping review are available in the manuscript or on request from the corresponding author. Data sharing is not applicable as no new datasets were generated.

## Abbreviations

APA: American psychological association
HMIC: Health Management Information Consortium
JBI: Joanna Briggs Institute
NHS: National Health Service
PRISMA-ScR: Preferred Reporting Items for Systematic Reviews and Meta-Analyses extension for Scoping Reviews
SPP: Social Policy and Practice
