# Supplementary material for: Live-in care for older people with a focus on those living with dementia: a scoping review
Source: BMC Geriatr. 2026 Jul 29;26:1346. doi: 10.1186/s12877-026-07983-w (PMC13700235; doi:10.1186/s12877-026-07983-w)
Supplement: Supplementary file 1 [file 12877_2026_7983_MOESM1_ESM.docx]

# Appendix 1

Search strategies

**Search strategies**

**Ovid MEDLINE(R) ALL <1946 to April 24, 2025>**

1	Nursing, Private Duty/

2	Home Health Nursing/

3	home care.ti,kw.

4	Live in care.ti,ab.

5	(professional care* adj5 home).ti,ab.

6	(formal care* adj5 home).ti,ab.

7	(carer* adj3 "live in").ti,ab.

8	domiciliary care.ti,ab.

9	personal care assistant*.ti,ab.

10	live-in caregiver*.ti,ab.

11	resident caregiver*.ti,ab.

12	resident carer*.ti,ab.

13	(shared lives or homeshare or "right at home").ti,ab.

14	or/1-13

15	elderly.ti,ab.

16	old*.ti,ab.

17	dement*.ti,ab.

18	alzheimer*.ti,ab.

19	frail*.ti,ab.

20	senior*.ti,ab.

21	geriatric*.ti,ab.

22	veteran*.ti,ab.

23	or/15-22

24	own home*.ti,ab.

25	personal home*.ti,ab.

26	at home.ti,ab.

27	their home.ti,ab.

28	home based.ti,ab.

29	home setting*.ti,ab.

30	24 or 25 or 26 or 27 or 28 or 29

31	14 and 23 and 30

*Checked Home aide

**Embase <1974 to 2025 April 28>**

1	home care.ti,kw.

2	Live in care.ti,ab.

3	(professional care* adj5 home).ti,ab.

4	(formal care* adj5 home).ti,ab.

5	(carer* adj3 "live in").ti,ab.

6	domiciliary care.ti,ab.

7	personal care assistant*.ti,ab.

8	live-in caregiver*.ti,ab.

9	resident caregiver*.ti,ab.

10	resident carer*.ti,ab.

11	(shared lives or homeshare or "right at home").ti,ab.

12	or/1-11

13	elderly.ti,ab.

14	old*.ti,ab.

15	dement*.ti,ab.

16	alzheimer*.ti,ab.

17	frail*.ti,ab.

18	senior*.ti,ab.

19	geriatric*.ti,ab.

20	veteran*.ti,ab.

21	or/13-20

22	own home*.ti,ab.

23	personal home*.ti,ab.

24	at home.ti,ab.

25	their home.ti,ab.

26	home based.ti,ab.

27	home setting*.ti,ab.

28	or/22-27

29	12 and 21 and 28

**APA PsycInfo <1806 to April 2025 Week 3>**

1	Live in care.ti,ab.

2	(professional care* adj5 home).ti,ab.

3	(formal care* adj5 home).ti,ab.

4	(carer* adj3 "live in").ti,ab.

5	domiciliary care.ti,ab.

6	personal care assistant*.ti,ab.

7	live-in caregiver*.ti,ab.

8	resident caregiver*.ti,ab.

9	resident carer*.ti,ab.

10	(shared lives or homeshare or "right at home").ti,ab.

11	or/1-10

12	elderly.ti,ab.

13	old*.ti,ab.

14	dement*.ti,ab.

15	alzheimer*.ti,ab.

16	frail*.ti,ab.

17	senior*.ti,ab.

18	geriatric*.ti,ab.

19	veteran*.ti,ab.

20	or/12-19

21	11 and 20

**HMIC Health Management Information Consortium <1979 to January 2025/Social Policy & Practice**

1	Live in care.ti,ab.

2	(professional care* adj5 home).ti,ab.

3	(formal care* adj5 home).ti,ab.

4	(carer* adj3 "live in").ti,ab.

5	domiciliary care.ti,ab.

6	personal care assistant*.ti,ab.

7	live-in caregiver*.ti,ab.

8	resident caregiver*.ti,ab.

9	resident carer*.ti,ab.

10	(shared lives or homeshare or "right at home").ti,ab.

11	or/1-10

12	elderly.ti,ab.

13	old*.ti,ab.

14	dement*.ti,ab.

15	alzheimer*.ti,ab.

16	frail*.ti,ab.

17	senior*.ti,ab.

18	geriatric*.ti,ab.

19	veteran*.ti,ab.

20	or/12-19

21	11 and 20

**Additional terms searched on MEDLINE, Embase and PsycINFO (September 2025):**

1	"24/7".ti,ab.

2	"24 hour".ti,ab.

3	"round the clock".ti,ab.

4	1 or 2 or 3

5	care.ti,ab.

6	"at home".ti,ab.

7	"own home".ti,ab.

8	private residence*.ti,ab.

9	domestic home*.ti,ab.

10	6 or 7 or 8 or 9

11	4 and 5 and 10

**Cinahl**

| **#** | **Query** |
| --- | --- |
| S28 | S19 AND S26 AND S27 |
| S27 | S1 OR S2 OR S3 OR S4 OR S5 OR S6 OR S7 OR S8 OR S9 OR S10 |
| S26 | S20 OR S21 OR S22 OR S23 OR S24 OR S25 |
| S25 | XB "home setting" |
| S24 | XB "home based" |
| S23 | XB "their home" |
| S22 | XB "at home" |
| S21 | XB "personal home*" |
| S20 | XB "own home*" |
| S19 | S11 OR S12 OR S13 OR S14 OR S15 OR S16 OR S17 OR S18 |
| S18 | XB veteran* |
| S17 | XB geriatric* |
| S16 | XB senior* |
| S15 | XB frail* |
| S14 | XB alzheimer* |
| S13 | XB dement* |
| S12 | XB old* |
| S11 | XB elderly |
| S10 | XB ("shared lives" or homeshare or "right at home") |
| S9 | XB "resident carer*" |
| S8 | XB "resident caregiver*" |
| S7 | XB "live in caregiver*" |
| S6 | XB "personal care assistant*" |
| S5 | XB "domiciliary care" |
| S4 | XB carer* N3 "live in" |
| S3 | XB "formal care*" N4 home |
| S2 | XB "professional care*" N4 home |
| S1 | XB "live in care" |

**Ageline**

| **#** | **Query** |
| --- | --- |
| S19 | S17 AND S18 |
| S18 | S11 OR S12 OR S13 OR S14 OR S15 OR S16 |
| S17 | S1 OR S2 OR S3 OR S4 OR S5 OR S6 OR S7 OR S8 OR S9 OR S10 |
| S16 | "home setting" |
| S15 | "home based" |
| S14 | "their home" |
| S13 | "at home" |
| S12 | "personal home*" |
| S11 | "own home*" |
| S10 | "shared lives" or homeshare or "right at home" |
| S9 | "resident carer*" |
| S8 | "resident caregiver*" |
| S7 | "live in caregiver*" |
| S6 | "personal care assistant*" |
| S5 | "domiciliary care" |
| S4 | carer* N3 "live in" |
| S3 | "formal care*" N4 home |
| S2 | XB "professional care*" N4 home |
| S1 | "live in care" |

**British Nursing Index**

| **Select all** | [**Set**](https://www.proquest.com/recentsearches.recentsearchtabview.recentsearchesgridview:toggellistorder?site=britishnursingindexft&t:ac=RecentSearches) | **Search** |
| --- | --- | --- |
| Select item 13 | **S13** | [[S1] OR [S2] OR [S4] OR [S5] OR [S6] OR [S7] OR [S8] OR [S9] OR [S10] OR [S11] OR [S12]](https://www.proquest.com/recentsearches.recentsearchtabview.recentsearchesgridview.scrolledrecentsearchlist.checkdbssearchlink:rerunsearch/6912728D71BF43CBPQ/None/$N?site=britishnursingindexft&t:ac=RecentSearches) |
| Select item 12 | **S12** | [abstract("shared lives" or homeshare or "right at home") OR title("shared lives" or homeshare or "right at home")](https://www.proquest.com/recentsearches.recentsearchtabview.recentsearchesgridview.scrolledrecentsearchlist.checkdbssearchlink:rerunsearch/5A0AA7747F814C24PQ/None/$N?site=britishnursingindexft&t:ac=RecentSearches) |
| Select item 11 | **S11** | [abstract("resident carer*") OR title("resident care*")](https://www.proquest.com/recentsearches.recentsearchtabview.recentsearchesgridview.scrolledrecentsearchlist.checkdbssearchlink:rerunsearch/B5243F1E7BF34C71PQ/None/$N?site=britishnursingindexft&t:ac=RecentSearches) |
| Select item 10 | **S10** | [abstract("resident caregiver*") OR title("resident caregiver*")](https://www.proquest.com/recentsearches.recentsearchtabview.recentsearchesgridview.scrolledrecentsearchlist.checkdbssearchlink:rerunsearch/F15B3CC1652F4A3APQ/None/$N?site=britishnursingindexft&t:ac=RecentSearches) |
| Select item 9 | **S9** | [abstract("live in caregiver*") OR title("live in caregiver*")](https://www.proquest.com/recentsearches.recentsearchtabview.recentsearchesgridview.scrolledrecentsearchlist.checkdbssearchlink:rerunsearch/E42CE6DE8B944714PQ/None/$N?site=britishnursingindexft&t:ac=RecentSearches) |
| Select item 8 | **S8** | [abstract("personal care assistant*") OR title("personal care assistant*")](https://www.proquest.com/recentsearches.recentsearchtabview.recentsearchesgridview.scrolledrecentsearchlist.checkdbssearchlink:rerunsearch/F153692841D044CFPQ/None/$N?site=britishnursingindexft&t:ac=RecentSearches) |
| Select item 7 | **S7** | [abstract("domiciliary care*") OR title("domiciliary care*")](https://www.proquest.com/recentsearches.recentsearchtabview.recentsearchesgridview.scrolledrecentsearchlist.checkdbssearchlink:rerunsearch/2AC61B7735F4DD7PQ/None/$N?site=britishnursingindexft&t:ac=RecentSearches) |
| Select item 6 | **S6** | [abstract(carer* N/3 "live in ") OR title(carer* N/3 "live in ")](https://www.proquest.com/recentsearches.recentsearchtabview.recentsearchesgridview.scrolledrecentsearchlist.checkdbssearchlink:rerunsearch/D23C7974FD904399PQ/None/$N?site=britishnursingindexft&t:ac=RecentSearches) |
| Select item 5 | **S5** | [abstract(carer*" NEAR/3 "live in") OR title(carer*" NEAR/3 "live in")](https://www.proquest.com/recentsearches.recentsearchtabview.recentsearchesgridview.scrolledrecentsearchlist.checkdbssearchlink:rerunsearch/D2A1E84049CB4B9DPQ/None/$N?site=britishnursingindexft&t:ac=RecentSearches) |
| Select item 4 | **S4** | [abstract(("formal care") N/5 home) OR title(("formal care") N/5 home)](https://www.proquest.com/recentsearches.recentsearchtabview.recentsearchesgridview.scrolledrecentsearchlist.checkdbssearchlink:rerunsearch/5CF1D8F2194D4EF9PQ/None/$N?site=britishnursingindexft&t:ac=RecentSearches) |
| Select item 2 | **S2** | [abstract("professional care*" N/5 home) OR title("professional care*" N/5 home)](https://www.proquest.com/recentsearches.recentsearchtabview.recentsearchesgridview.scrolledrecentsearchlist.checkdbssearchlink:rerunsearch/5F9CCBA78B9F42ACPQ/None/$N?site=britishnursingindexft&t:ac=RecentSearches) |
| Select item 1 | **S1** | [abstract("live in care*") OR title("live in care*")](https://www.proquest.com/recentsearches.recentsearchtabview.recentsearchesgridview.scrolledrecentsearchlist.checkdbssearchlink:rerunsearch/545C89B065B74CD1PQ/None/$N?site=britishnursingindexft&t:ac=RecentSearches) |

**ASSIA/Sociology Database/Sociological Abstract/ProQuest Dissertations & Theses**

[(abstract("live in care*") OR title("live in care*")) OR (abstract("professional care*" NEAR/5 home) OR title("professional care*" NEAR/5 home)) OR (abstract(("formal care") NEAR/5 home) OR title(("formal care") NEAR/5 home)) OR (abstract(carer* " NEAR/3 " live in "") OR title(carer* " NEAR/3 " live in "")) OR (abstract(carer* NEAR/3 "live in ") OR title(carer* NEAR/3 "live in ")) OR (abstract("domiciliary care*") OR title("domiciliary care*")) OR (abstract("personal care assistant*") OR title("personal care assistant*")) OR (abstract("live in caregiver*") OR title("live in caregiver*")) OR (abstract("resident caregiver*") OR title("resident caregiver*")) OR (abstract("resident carer*") OR title("resident care*")) OR (abstract("shared lives" OR homeshare OR "right at home") OR title("shared lives" OR homeshare OR "right at home"))](https://www.proquest.com/recentsearches.recentsearchtabview.recentsearchesgridview.scrolledrecentsearchlist.checkdbssearchlink:rerunsearch/B3F33ECE02D24047PQ/None/$N?site=assia&t:ac=RecentSearches)

**Cochrane Library**

Search Name:	Live in care

Date Run:	01/05/2025 15:13:41

Comment:

ID	Search	Hits

#1	MeSH descriptor: [Nursing, Private Duty] explode all trees

#2	MeSH descriptor: [Home Health Nursing] explode all trees

#3	"Live in care":ti,ab

#4	((professional NEXT care) NEAR/5 (home)):ti,ab

#5	((formal NEXT care*) NEAR/5 (home)):ti,ab

#6	((carer*) NEAR/3 (live NEXT in)):ti,ab

#7	"domiciliary care":ti,ab

#8	(personal NEXT care NEXT assistant*):ti,ab

#9	("live-in" NEXT caregiver*):ti,ab

#10	(resident NEXT caregiver*):ti,ab

#11	(resident NEXT carer*):ti,ab

#12	("shared lives" or homeshare or "right at home"):ti,ab

#13	(home NEXT aide*):ti,ab

#14	(inhouse NEXT carer*):ti,ab

#15	(inhouse NEXT caregiver*):ti,ab

#16	"dwell in care":ti,ab

#17	#1 or #2 or #3 or #4 or #5 or #6 or #7 or #8 or #9 or #10 or #11 or #12 or #13 or #14 or #15 or #16

#18	"at home":ti,ab

#19	"their home":ti,ab

#20	"home based":ti,ab

#21	(home NEXT setting*):ti,ab

#22	"own home":ti,ab

#23	"personal home":ti,ab

#24	#18 or #19 or #20 or #20 or #21 or #22 or #23

#25	#17 and #24

**Social Science Citation Index**

TS=("live in care*")

**CPCI-SSH**

TI=“live-in care*”

**Grey Literature**

*“live in care”* 1^st^ May 2025

WHO Institutional Repository:

<https://www.kent.ac.uk/library-it/your-digital-library/digital-resources-directory/404>

WorldCat

CORE (Collection of Open Access Research)

Google Scholar

Bielefeld Academic Search Engine

**Websites** 2^nd^ May 2025

*‘Live in care’*

- Age UK
- Alzheimer’s Association (US)
- Alzheimer’s Society (UK)
- Alzheimer’s Disease International
- Homecare Association (UK)

Searched through Research and reports page for publications/models of care

•	County council websites (N=29)

**Hand-searching journals** (1 year + title searches)

Age and Ageing

Ageing and Mental Health

Ageing and Society

*BMC Geriatrics*

Journal of Long Term Care

Health and Social care in the Community

BMJ Supportive and palliative care

Home Health Care Services Quarterly
